# Rational numbers: A systematic review and ALE meta-analysis of the neuroimaging of fraction and decimal processing in the brain

**DOI:** 10.3758/s13423-026-02939-y

**Published:** 2026-06-15

**Authors:** Isabella Starling-Alves, Danielle Ann S. Bonilla, Sasha B. Bandler, Eric D. Wilkey

**Affiliations:** 1 Department of Educational Psychology, College of Education and Human Sciences, University of Nebraska–Lincoln, Lincoln, NE USA; 2 Department of Psychology and Human Development, Peabody College, Vanderbilt University, 230 Appleton Place, Nashville, TN 37203 USA

**Keywords:** Rational number processing, Fractions, Decimals, FMRI, Neuroimaging, IPS, ALE meta-analysis

## Abstract

Mathematics knowledge is crucial for success in our society. Among the various mathematical areas, rational number knowledge is particularly significant for academic achievement. However, rational number knowledge is challenging, and many children and adults struggle with tasks involving fraction and decimal magnitude processing. In this study, we conducted a systematic review of functional magnetic resonance imaging (fMRI) studies to identify brain regions associated with fractions and decimals. Additionally, we synthesized fraction studies in an activation likelihood estimation (ALE) meta-analysis. A descriptive synthesis of studies showed that fractions and decimals are both associated with activation in the intraparietal sulcus and frontal regions. However, multivariate findings suggest that their neural representation is dissimilar. In addition, the ALE meta-analysis indicated that the IPS, the middle frontal gyrus, the middle temporal gyrus, and the precuneus are associated with fraction processing. These regions have been associated with higher order cognition, including inhibitory control and working memory, and nonsymbolic magnitude processing in previous studies. We discuss current limitations of the field, such as the small number of studies targeting decimal processing, suggesting directions for future studies.

**Supplementary Information:**

The online version contains supplementary material available at 10.3758/s13423-026-02939-y.

## Introduction

Imagine you are grocery shopping and trying to decide which option gives you the best deal for strawberries: ¼ lb for $1.50 or ⅓ lb for $1.90. How would you solve this problem? While this may seem like a simple calculation, many people struggle with comparing rational numbers. For example, someone might incorrectly choose ¼ lb for $1.50 (equivalent to $6.00 per pound) over ⅓ lb for $1.90 ($5.70 per pound) by mistakenly believing that ¼ is greater than ⅓ and inferring that they are getting a cheaper price for a greater portion. Such errors in judging the magnitudes of rational numbers are well documented in the literature, even among adults with high levels of education (e.g., Gabriel et al., 2013; Schneider & Siegler, 2010). Yet the underlying reasons for these persistent difficulties remain unclear. Gaining a deeper understanding of the neural and cognitive mechanisms involved in rational number processing can help uncover why these challenges occur and guide the design of effective educational interventions. To contribute to this understanding, this systematic review of functional magnetic resonance imaging (fMRI) studies examines the neural systems that support rational number processing, with a particular focus on fractions and decimals.

### *Rational number knowledge*

**Fig. 1:**
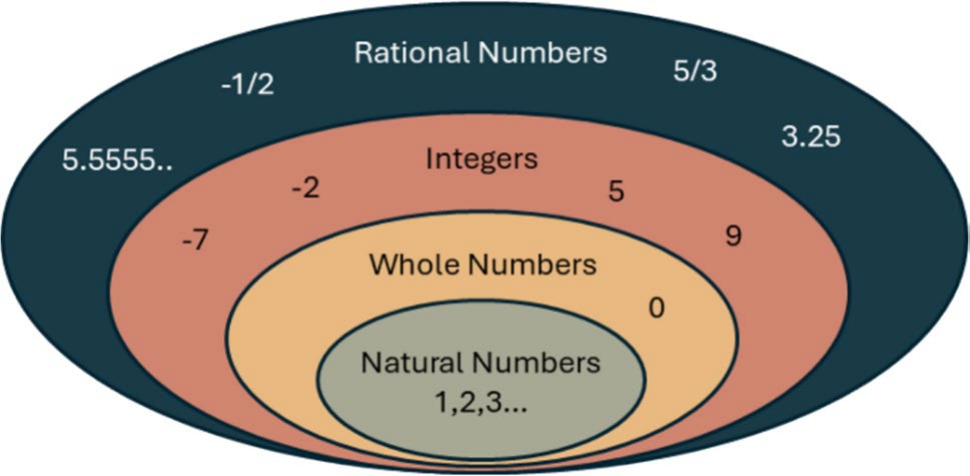
Number sets encompassed by rational numbers. This figure illustrates various number sets, including natural numbers, whole numbers, integers, and rational numbers. It shows that all natural numbers, whole numbers, and integers are subsets of rational numbers. However, some rational numbers, such as fractions and decimals, do not belong to the other number sets

*Rational number knowledge* can be defined as the ability to understand rational number concepts (e.g., magnitude and density) and perform arithmetic operations (e.g., addition, subtraction, multiplication, and division) with rational numbers (Bailey et al., 2014; Booth & Newton, 2012). Rational numbers are those that express a relation between magnitudes and can be written in the form a/b, where b ≠ 0. This broad category encompasses natural numbers, whole numbers, integers, fractions, decimals, proportions, percentages, and other expressions (see Fig. 1). In this study, however, we use the term *rational number* knowledge more narrowly to refer specifically to knowledge of *fractions* (e.g., ½) and *decimals* (e.g., 0.5)*.*

Rational numbers are widely recognized as one of the most challenging topics in the elementary school curriculum, being described as a gatekeeper for higher-level mathematics (Booth & Newton, 2012; Fazio & Siegler, 2011; Lortie-Forgues et al., 2015). Difficulties with fractions and decimals are not merely incidental but often persist into adulthood. For example, even college students make errors when solving rational number problems, such as comparing the magnitudes of fractions and decimals and solving fraction operations with unlike terms (Lee & Boyadzhiev, 2020; Schneider & Siegler, 2010). Moreover, many preservice mathematics teachers face challenges in conceptual and procedural fraction knowledge—for instance, failing to describe what it means to divide a fraction by another fraction and solve fraction division operations (Lo & Luo, 2012; Ma, 1999; Steenbrugge et al., 2014). These persistent challenges are particularly concerning because rational number knowledge has been identified as a strong predictor of success in advanced mathematics, including algebra (Barbieri et al., 2021; Booth et al., 2014; DeWolf et al., 2015; Hurst & Cordes, 2018; Liang et al., 2018; Wu, 2001). In other words, students who struggle with fractions and decimals in the early grades may face cumulative disadvantages, as these skills form a critical foundation for later mathematical learning and, by extension, for many academic and career opportunities that depend on strong quantitative reasoning.

### Cognitive mechanisms that influence mastery of rational numbers knowledge

To understand why acquiring rational number knowledge is challenging, researchers have targeted underlying cognitive mechanisms (Siegler & Lortie-Forgues, 2017). Three primary, yet interconnected, hypotheses have been proposed. One hypothesis, which we refer to as the *lack of grounding hypothesis*, suggests that humans struggle with rational numbers due to a lack of a perceptual system that can ground these numerical symbols (Feigenson et al., 2004). This hypothesis assumes that humans have an intuitive system dedicated to processing nonsymbolic absolute magnitudes (i.e., the approximate number system), such as sets of dots, which supports the understanding of symbolic numbers (Halberda et al., 2008; Piazza, 2011). However, this perceptual system may not specialize to nonsymbolic ratio magnitudes, which makes the semantics of rational numbers disconnected from intuitive quantity representations. In the absence of a direct perceptual basis, rational numbers become highly abstract, posing significant challenges for conceptual understanding.

Another explanation attributes difficulties with rational number knowledge to their inherent complexity. We refer to this hypothesis as the *cognitive load hypothesis* (Lortie-Forgues et al., 2015). This account proposes that understanding a rational number requires integrating information from its components—such as numerators and denominators in fractions or the whole number and decimal parts in decimals—to form a holistic sense of its magnitude. This integration process places high demands on limited attentional resources. When these resources become overloaded, processing rational numbers becomes slower, less accurate, and more error-prone.

Finally, the *natural number bias hypothesis* assumes that difficulties with rational numbers stem from an interference from preexisting natural number knowledge (Ni & Zhou, 2005). By the time students are introduced to rational numbers in school, they have accumulated years of experience and practice with natural numbers. As a result, they may have automatized certain heuristics for natural numbers that do not hold for rational numbers—for example, that larger digits represent larger magnitudes, multiplication always increases magnitude, and division always decreases magnitude. When working with rational numbers, this preexisting natural number knowledge may be automatically activated, creating interference. Successfully processing rational numbers therefore may require inhibitory control to suppress these natural number-based heuristics and apply rational number-specific reasoning instead (Leib et al., 2023; Ni & Zhou, 2005; Van Hoof et al., 2020). This interference, combined with the demand for inhibitory control, can pose significant challenges for rational number tasks.

While these hypotheses highlight distinct mechanisms, they converge on the idea that rational number processing is more cognitively demanding than natural number processing. Importantly, these hypotheses are not mutually exclusive as multiple mechanisms may operate simultaneously. For example, a lack of grounding might prompt individuals to default to natural number representations when solving rational number problems, and the need to inhibit these preexisting representations could, in turn, increase cognitive load. The interplay among these distinct cognitive mechanisms suggests that rational numbers recruit more cognitive resources relative to other numerical representations. Behavioral evidence has supported this view by showing lower accuracy and speed in rational number tasks than whole number tasks, along with associations between executive mechanisms and rational number processing (e.g., Avgerinou & Tolmie, 2020; Hurst & Cordes, 2016; Leib et al., 2023; Roell et al., 2017). However, in recent years, neuroimaging studies investigating rational number processing have increased (Obersteiner et al., 2019; Rosenberg-Lee, 2021). These studies complement behavioral findings by showing how brain regions involved in fraction and decimal processing compare with those associated with other number formats, including natural numbers and nonsymbolic magnitudes.

### Advancing rational number research through neuroimaging

Neuroimaging studies can identify the brain regions and networks involved in rational number processing, providing insights into the specific and shared mechanisms of fraction and decimal processing. Obersteiner and colleagues (2019) integrated findings from several neuroimaging studies and reported that fraction processing is consistently associated with activity in the intraparietal sulcus (IPS). Expanding this scope, Rosenberg-Lee (2021) reviewed neuroimaging studies on nonsymbolic ratios, fractions, and decimals. This study confirmed the role of the IPS across a broader range of rational number tasks and suggested that such tasks may also recruit additional cognitive functions, such as inhibitory control. Together, these reviews provide a valuable overview of the neural correlates of rational number processing, consistently highlighting the involvement of the IPS and indicating that rational numbers recruit different resources relative to natural numbers. However, previous studies have not systematically analyzed differences between neurocognitive systems associated with fractions, decimals, and natural numbers. A systematic review of neuroimaging studies on rational number processing can provide more rigorous, transparent, and replicable findings than nonsystematic reviews.

### Current study

In the current study, we conducted a systematic review to identify brain regions associated with rational number processing. In particular, we investigate brain regions linked to (1) fraction processing and (2) decimal processing. We integrate findings from fraction and decimal functional magnetic resonance (fMRI) studies, and follow up with an investigation of the convergence and divergence of foci associated with fraction processing via an activation likelihood estimation (ALE) meta-analysis. Unlike prior reviews (e.g., Obersteiner et al., 2019; Rosenberg-Lee, 2021), our study systematically synthesizes the neuroimaging evidence on fractions and decimals, integrates findings about fractions through an ALE meta-analysis, and discusses methodological limitations in the literature. With this approach, we provide a more rigorous overview of fMRI studies, identifying brain regions involved in rational number processing. These findings may inform current cognitive theories and guide future research, including the identification of potential regions of interest for neuroimaging investigations.

## Methods

To investigate the brain regions associated with rational number processing, we integrated findings from journal and conference articles, preprints, theses, and dissertations that examined children’s and adults’ fraction or decimal processing skills using functional magnetic resonance imaging (fMRI). We did not impose constraints on these distinct developmental stages, as characterizing neural correlates in both children and adults provides a more comprehensive understanding of rational number processing and allows identification of patterns that may be shared or differ between these groups.

After searching a diverse set of databases, we screened and selected studies for inclusion. The first, second, and third authors conducted data screening and extraction independently in COVIDENCE, a web-based tool that streamlines the systematic review process, following the PRISMA guidelines (Page et al., 2021). The authors discussed any screening or extraction disagreements between them and, if no agreement was reached, the fourth author resolved the matter. We then integrated findings from these studies and assessed their quality. In addition, we identified a subset of studies that reported brain coordinates and conducted an ALE meta-analysis to examine convergence in the reported foci associated with rational number processing. ALE is a coordinate-based meta-analytic procedure that treats reported foci as spatial probability distributions centered on the coordinates. It then computes the union of activation across voxels and contrasts this with random noise using permutation procedures (Eickhoff et al., 2009; Müller et al., 2018). The following sections describe the systematic review search strategy, eligibility criteria, and screening and extraction procedures, as well as the ALE meta-analysis methods.

### Databases and search strategy

We conducted our search iteratively in July 2023 across the following databases: Web of Science, PubMed, MedLine, PsycINFO, ProQuest Theses and Dissertations, and PsyArXiv. We used the following search terms without any filters:

((("Decimal") OR ("Fraction comparison") OR ("Fraction processing") OR ("Fraction understanding") OR ("Fraction knowledge") OR ("Fraction magnitude") OR ("Ratio comparison") OR ("Ratio processing") OR ("Ratio understanding”) OR ("Ratio knowledge") OR ("Ratio magnitude") OR ("Proportional reasoning") OR ("Rational number")) AND (("fMRI") OR ("MRI") OR ("magnetic resonance imaging") OR (neuroimag*) OR ("brain")))

We selected these filters to capture a broad range of rational number skills. After screening the results based on their title and abstract, we further expanded our search by conducting a forward and backward search. All searches were conducted in July 2023, with no restrictions on initial publication date. Consistent with standard systematic review procedures, the present study reflects the state of the literature as of this predefined search cutoff date. Maintaining a fixed search window ensures transparency, reproducibility, and methodological consistency of the study selection and ALE meta-analysis. More recent studies published after the search cutoff provide important additional evidence but were not included in the formal analyses to preserve the integrity of the predefined search and analysis protocol (Page et al., 2021). This approach consists of identifying relevant studies that cited the preselected studies (i.e., forward step; conducted using Google Scholar) or were cited by them (i.e., backward step; done by examining the reference lists of the preselected articles to identify additional studies).

### Eligibility criteria

We included empirical studies that used fraction or decimal tasks (e.g., comparison, estimation) and reported fMRI results. Eligible publication types comprised journal and conference articles, preprints, theses, and dissertations. Papers not published in English were excluded due to limited resources for translation. We further excluded: (1) conference abstracts, because they did not provide sufficient methodological and results details; (2) review articles, because they did not present the primary empirical data needed to address our research questions; (3) studies that assessed only nonsymbolic ratio processing or whole-number processing; (4) studies that did not use fMRI; and (5) studies with atypical populations.

### Screening

We initially screened the studies based on their title and abstract. We preselected those studies with titles and abstracts mentioning rational number processing tasks and thoroughly read them, applying the inclusion and exclusion criteria. This process allowed us to identify the studies that used neuroimaging methods and subsequently underwent the extraction protocol.

### Extraction

The extraction protocol covered various aspects of the studies, including title, authors, leading author contact, funding sources, conflicts of interest, the country where the study was conducted, and studies’ aims, design, and main results. Participant characteristics included initial and final sample size, participants’ age, gender, grade, recruitment procedures, and inclusion and exclusion criteria. Regarding the measures, we extracted information about the number processing task (e.g., comparison, estimation, arithmetic operation), the type of rational number (e.g., fractions or decimals), the magnitudes used in the task (e.g., 0.5, 0.6, 0.7), and whether the study included a measure of integer number processing. For imaging parameters, we documented the MRI scanner model and acquisition settings. From the results, we extracted information on participants’ response time and accuracy in the ratio processing tasks when available. We also extracted the fMRI contrasts analyzed, associated peak activation coordinates, cluster sizes, reported effect sizes, and coordinate system used to report results. No additional information was sought from authors. Although contacting authors to request additional or unpublished contrasts could potentially increase the number of eligible experiments, we chose to restrict our review to publicly available data to ensure transparency, reproducibility, and consistency across included studies. We share the extraction protocol on OSF, along with additional information extracted for analytical procedures, such as list of coordinates (https://osf.io/gf254/?view_only=2a30366d355b4486ae74d7e18404bb76).

### ALE meta-analysis

We conducted an exploratory ALE meta-analysis (Eickhoff et al., 2009; Turkeltaub et al., 2012) to identify convergent activation related to fraction and decimal processing, based on findings from task-based fMRI studies. In this analysis, we included extracted coordinates from a subset of studies that reported whole-brain univariate analyses targeting fraction processing.

When conducting ALE meta-analyses, it is recommended to balance the inclusion of more studies to maximize statistical power with restricting studies to maintain methodological homogeneity (Müller et al., 2018). To achieve this balance, we first ran an analysis that included a broad set of contrasts to increase the number of eligible studies. These contrasts encompassed the *easy versus hard* comparisons in fraction tasks (i.e., manipulated as the distance between the fractions being compared) and *fraction versus decimal*, *fraction versus natural number*, or *fraction versus nonsymbolic magnitude*. In these analyses, each study was counted once when reporting the number of included studies, even if multiple contrasts or experiments were reported. We then conducted a sensitivity analysis (i.e., leave-one-study-out sensitivity analysis) to investigate how each study contributed to findings (Leroy et al., 2020; Wilson et al., 2018).

We conducted these analyses as within-study comparisons, using BrainMap GingerALE (Version 3.0.2). Coordinates reported in Talairach space were converted to MNI space using BrainMap’s *icbm2tal* transform prior to analysis. Following recommendations for ALE meta-analyses (Eickhoff et al., 2016), we applied a cluster-level family-wise error (FWE) correction at *p* <.05, with a voxel-level threshold of *p* <.001 and 1,000 permutations. We used MRIcroGL for data visualization (Rorden, 2025), and the anatomical labels generated by GingerALE software. When interpreting our findings, we operationalized *overlapping*, or *converging* regions as those consistently reported across independent experiments, identified through ALE convergence. We also used Neurosynth (neurosynth.org) to identify additional cognitive skills associated with the converging areas (i.e., MNI coordinates) reported in the analyses.

## Results

### Review process

**Fig. 2:**
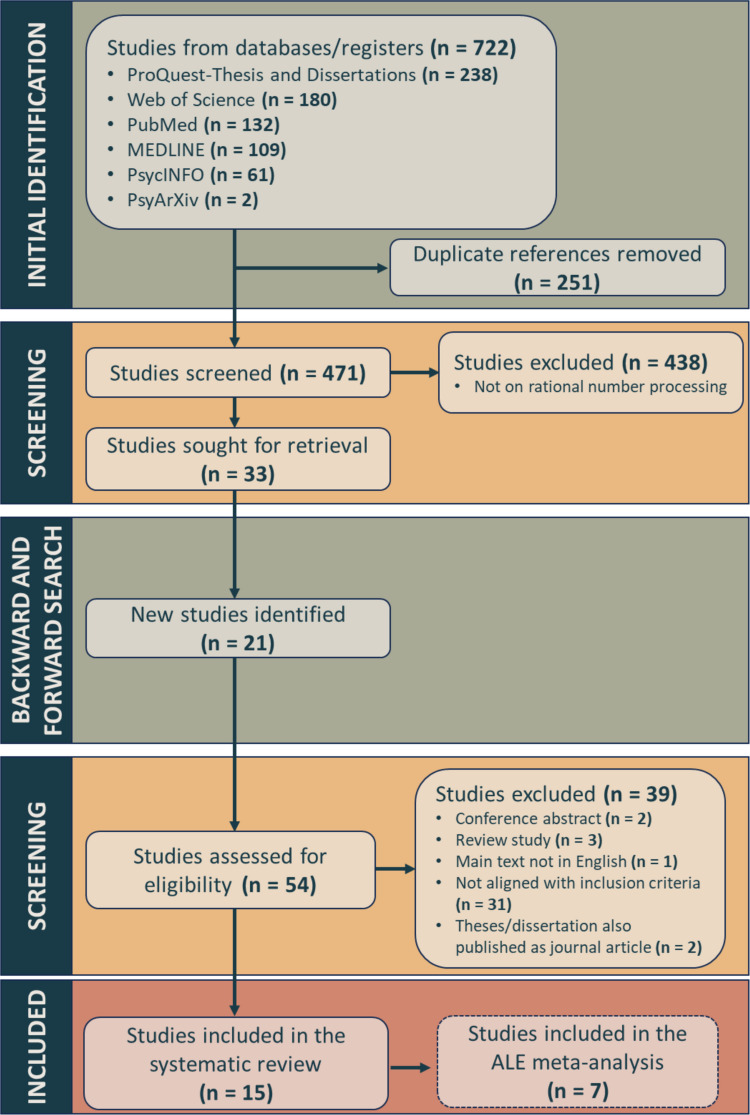
Flow diagram of study inclusion based on PRISMA

The review process is illustrated in Fig. 2. The initial search resulted in 722 studies. Out of these studies, 251 duplicates were identified and excluded by COVIDENCE. We screened the remaining 471 studies and preselected 33 relevant ones, which referred to fMRI methods and rational number processing measures in their titles and abstracts. Through backward and forward searches in these relevant studies, 21 additional studies were included in our sample. We then read these 54 studies in full. We excluded a total of 37 studies that did not meet the inclusion criteria: two conference abstracts, three review papers, one manuscript in French, and 31 studies whose methods did not correspond to our inclusion criterion (i.e., 22 that did not report fMRI data, five that only used tasks with integer numbers, two that only used nonsymbolic magnitudes, one that used irrational numbers, and one that targeted participants with fragile X). When a thesis or dissertation reported overlapping data with a peer-reviewed paper or preprint, we prioritized the manuscript version. Thus, we further excluded two dissertations (i.e., DeWolf, 2016; Park, 2021), since their results were also reported in other selected articles. After exclusion, 15 empirical studies were included for the review and underwent the extraction protocol.

### Characteristics of the included publications

Among the 15 studies, there were 10 journal articles, three thesis/dissertations, one preprint, and one conference article. One of the selected studies (Park et al., 2023) was originally published as a preprint at the time of the search procedure, but was later published in a journal (Park et al., 2025). We conducted analyses based on the preprint to keep consistency with our extraction procedure described above. As shown in Table 1, most studies targeted only adult populations (*n* = 13 studies), informing on the brain regions associated with more advanced rational number knowledge, but not earlier developmental stages. This pattern highlights the need for more neuroimaging studies investigating developmental trajectories of rational number processing in children and adolescents.

**Table 1:** Characteristics of included studies (organized by authors’ name alphabetical order)

Reference	Study type	Sample	Measured natural number processing?	Brief description of experimental paradigm analyzed in this review	Trials	Analytical methods
Bhatia et al., 2022	Journal Article	Adults (*n* = 48)	Yes	Task 1: Adaptation to: nonsymbolic absolute magnitudes (lines), natural numbers (single digits and two digits), nonsymbolic ratio magnitudes (line ratios), and fractions	640	Univariate analyses and MVPA (RSA)
Binzak, 2020	Thesis or Dissertation	Adults (*n* = 24)	No	Task 1: Fraction vs. fraction comparison; fraction vs. nonsymbolic magnitude comparison; nonsymbolic magnitude vs. nonsymbolic magnitude comparison	216	Univariate analyses
Cui et al., 2020*	Journal Article	Adults (*n* = 68)	Yes	Task 1: Fraction vs. fraction comparison	128	Univariate analyses and MVPA (searchlight analysis)
DeWolf et al., 2016*	Journal Article	Adults (*n* = 16)	Yes	Task 1: Fraction vs. fraction comparison Task 2: Decimal vs. decimal comparison	240	Univariate analyses and MVPA (RSA)
Fassbender et al., 2014	Journal Article	Adults (*n* = 14)	No	Task 1: Decimal vs. decimal comparison (two conditions: only zeros after the decimal point or other numbers after decimal point)	62	Univariate analyses
Ischebeck et al., 2009*	Journal Article	Adults (*n* = 17)	No	Task 1: Fraction vs. fraction comparison	256	Univariate analyses
Jacob & Nieder, 2009	Journal Article	Adults (*n* = 12)	No	Task 1: Fraction (Arabic digits and number words) adaptation	360	Univariate analyses
Mock et al., 2018*	Journal Article	Adults (*n* = 24)	No	Task 1: Fraction vs. fraction comparison Task 2: Decimal vs. decimal comparison	96	Univariate analyses
Mock et al., 2019*	Journal Article	Adults (*n* = 24)	No	Task 1: Fraction vs. fraction comparison Task 2: Decimal vs. decimal comparison	96	Univariate analyses and MVPA (RSA)
Park et al., 2023*^º^	Journal Article	Children (*n* = 61)	No	Task 1: Fraction vs. fraction comparison; fraction vs. nonsymbolic magnitude comparison; nonsymbolic magnitude vs. nonsymbolic magnitude comparison	216	Univariate analyses and MVPA (RSA)
Schmithorst & Brown, 2004	Journal Article	Adults (*n* = 15)	No	Task 1: Fraction addition and subtraction	60	Independent component analysis
Sprute, 2013*	Thesis or Dissertation	Adults (*n* = 22–31)	Yes	Task 1: Adaptation to: fraction, decimals, and natural numbers (two digits) Task 2: Fraction vs. fraction comparison Task 3: Decimal vs. decimal comparison	Adaptation: 1752 Comparison: 324	Univariate analyses and MVPA (searchlight analysis)
Starling Alves, 2021	Thesis or Dissertation	Children (*n* = 38)	No	Task 1: Fraction vs. fraction comparison; fraction vs. nonsymbolic magnitude comparison; nonsymbolic Magnitude vs. nonsymbolic magnitude comparison	216	Univariate analyses
Tzur & Depue, 2014	Conference Article	Adults (*n* = 21)	Yes	Task 1: Fraction vs. fraction comparison	360	Univariate analyses
Wortha et al., 2020	Journal Article	Adults (*n* = 24)	No	Task 1: Fraction vs. fraction comparison	192	Univariate analyses

*RSA* representation similarity analysis; *Studies included in ALE meta-analysis; ºAlthough this study was later published as a peer-reviewed article, all analyses were conducted based on the preprint version available at the time of data extraction

Regarding the experimental methods, most fMRI paradigms consisted of comparison tasks (*n* = 14). These tasks were used to index the *neural distance effect* or to contrast neural activation patterns across number formats. At the behavioral level, it has been observed that people tend to show higher accuracy and faster response times when comparing numerical magnitudes separated by a far distance (e.g., 1 vs. 9, distance = 8) than by a near distance (e.g., 1 vs. 2, distance = 1; Moyer & Landauer, 1967). This pattern is known as the distance effect, which has been replicated across several developmental stages and cultures (e.g., Ashkenazi et al., 2009; Dehaene, 1996; Holloway & Ansari, 2009; Lukas et al., 2014). The distance effect, and the fact that it decreases with larger numerical magnitudes (together referred to as the ratio effect), is thought to reflect how numerical magnitudes are represented in the human brain: on a logarithmically compressed mental number line, extending from left to right (Dehaene, 2003; Nieder, 2016; though see Krajcsi et al., 2016; van Opstal & Verguts, 2011, for alternate explanations). At the neural level, the distance effect has been indexed through the neural distance effect: increased activation when comparing magnitudes separated by a near distance rather than a far distance, suggesting more effortful processing (Ansari et al., 2006; Piazza et al., 2004). Building on this effect, fMRI studies of rational numbers have contrasted near versus far distances in fractions and decimals as a proxy for magnitude processing, and have compared these distance effects—or overall activation patterns—with those elicited by whole numbers and nonsymbolic magnitudes.

Fractions were the most frequently targeted rational number format (*n* = 14), whereas only a few studies (*n* = 5) included decimals. In particular, among the selected studies, ten targeted only fractions (i.e., Bhatia et al., 2022; Binzak, 2020; Cui et al., 2020; Ischebeck et al., 2009; Jacob & Nieder, 2009; Park et al., 2023; Schmithorst & Brown, 2004; Starling Alves, 2021; Tzur & Depue, 2014; Wortha et al., 2020), four targeted both fractions and decimals (i.e., DeWolf et al., 2016; Mock et al., 2018, 2019; Sprute, 2013) and only one exclusively targeted decimals (i.e., Fassbender et al., 2014). These findings suggest that the literature has focused more on fraction processing, leaving the brain regions associated with decimal number processing underexplored.

### Quality of studies

Given the limited number of fMRI studies investigating fraction and decimal processing, we decided to synthesize all identified studies. However, their quality must be considered when interpreting the findings. To assess study quality, we examined publication type (i.e., whether the study was peer-reviewed), preregistration, sample size, and whether fMRI acquisition parameters, preprocessing pipeline, and analysis software were reported. Regarding the analyses, we considered the type of approach conducted (i.e., univariate contrasts, MVPA, or independent component analysis), the statistical thresholds applied, and whether reported coordinates included a clearly stated coordinate system. A table summarizing these characteristics of the selected studies is available on OSF (https://osf.io/gf254/?view_only=2a30366d355b4486ae74d7e18404bb76).

Among the 15 studies included, 12 were peer-reviewed. None of the studies reported preregistration, making it unclear whether the presented analyses fully aligned with the authors’ original hypotheses and analytical plans. Most studies relied on small sample sizes (range: 12–68, *mean* = 29.13), raising concerns about statistical power (Nee, 2019; Turner & Miller, 2013). All studies described their experimental design, but one did not report acquisition parameters, preprocessing pipeline, or analysis software (Tzur & Depue, 2014). Consequently, we placed less weight on this study when integrating findings.

All 15 studies conducted univariate analyses, with 13 reporting peak coordinates. One study (Starling Alves, 2021) reported primarily region-of-interest results, without extensively describing coordinates, and another (Tzur & Depue, 2014) reported only anatomical labels without specifying which atlas was used. Except for Tzur and Depue (2014), all studies reported both the thresholds used and the coordinate system. Thresholding approaches varied, with many studies applying uncorrected voxel-level *p* <.001 in combination with cluster-level FWE-corrected *p* <.05 or a minimum cluster size (*k* ranging from 10 to 67). In addition to univariate analyses, six studies employed MVPA. MVPA encompasses methods that analyze neural responses as distributed patterns of activity (Haxby, 2012; Norman et al., 2006). Unlike univariate approaches, which test each voxel independently, MVPA integrates information across multiple voxels to detect differences in distributed activation patterns (Mahmoudi et al., 2012).

Overall, the characteristics of the studies reported above highlights the need for fMRI studies with increased methodological rigor. Since most studies include smaller sample sizes (i.e., 11 studies had *n* < 30), their findings may lead to both false positives and false negatives (Szucs & Ioannidis, 2020). This limitation can be addressed with studies with larger sample sizes or within-subject designs that include a greater number of trial repetitions, increasing reliability (Turner & Miller, 2013). Furthermore, given current debates on replication and robustness in fMRI research (Bennett & Miller, 2013; Larsson et al., 2016; Nee, 2019; Yarkoni, 2009), the field would benefit from preregistered studies and the use of stricter statistical thresholds to avoid spurious findings (Eklund et al., 2016). These directions would increase transparency and robustness of findings. Finally, the adoption of analytical tools that complement univariate approaches, such as MVPA, can support testing hypotheses related to the identification of unique and overlapping brain regions across rational number formats.

### Broad summary of studies’ findings

First, we provide a brief description of the selected studies, qualitatively synthesizing their main findings. These findings are summarized in Supplementary Materials, Table S1.

#### Univariate analyses

First, we examined the brain regions more frequently identified as being associated with fraction processing and decimal processing. Altogether, studies using *univariate analysis* (i.e., voxel-wise comparisons) and relying on the distance effect have shown that frontal and parietal regions, including the superior parietal lobe around the posterior portion of the superior wall of the intraparietal sulcus, bilaterally, are associated with fraction and decimal processing (see Supplementary Materials, Table S1).These regions are also linked to natural number processing and nonsymbolic magnitude processing, suggesting they play a central role in numerical processing (Binzak, 2020; DeWolf et al., 2016; Jacob & Nieder, 2009; Mock et al., 2018, 2019; Park et al., 2023; Schmithorst & Brown, 2004; Sprute, 2013; Starling Alves, 2021; Tzur & Depue, 2014; Wortha et al., 2020).

We also explored differences between decimal and fraction processing. Mock and colleagues (2018) presented college students with fraction and decimal comparison tasks, in addition to nonsymbolic formats. Then, they conducted analyzes separated for each format, targeting the neural distance effect. Results indicated a greater number of regions associated with neural distance effect elicited by decimal comparison than fraction comparison. Specifically, decimal processing was linked with the bilateral IPS, and left-lateralized regions, including the left occipito-temporal cortex, left fusiform gyrus, left insula, and left inferior, middle, and superior frontal gyrus. Fraction processing was associated with the right IPS, the bilateral supplementary motor area, and the bilateral frontal gyrus. Thus, while activation within the right IPS was shared across these formats, they also elicited activity in distinct brain regions.

Other studies have corroborated differences across these formats. For instance, there is evidence that fractions elicit more activation than decimals in the superior and middle frontal gyri, the inferior and middle temporal gyri, the cingulate gyrus, and the cerebellum (DeWolf et al., 2016; Sprute, 2013). Using fraction and decimal comparison tasks, DeWolf and colleagues (2016) found that fraction processing elicited greater activation than decimal processing within the left IPS, the left precentral gyrus, the middle frontal gyrus, and the middle temporal gyrus. Authors interpreted their findings as evidence that fraction elicits higher cognitive load than decimals, possibly due to its bipartite structure. Therefore, their findings align with the cognitive load hypothesis.

Importantly, the brain regions recruited by fractions or decimals appear to depend on their specific features. For instance, Ischebeck and colleagues (2009) reported that comparing fractions with the same numerator (e.g., ⅓ vs. ¼) elicits greater activity in inferior frontal regions, the IPS, the supplementary motor area, and the occipital lobe than comparing fractions with the same denominator (e.g., ⅓ vs. ⅔). For decimals, Fassbender and colleagues (2014) showed that round decimals (e.g., 2.00) elicit increased activity within the nucleus accumbens relative to nonround decimals (e.g., 2.03). Taken together, these findings suggest that although fractions and decimals engage common brain regions such as the IPS, they also recruit distinct areas.

Then, we analyzed differences in representation, summarizing regions more closely linked with either fractions or decimals relative to natural numbers. Differences between fractions and natural numbers have been reported in the superior, middle, and inferior frontal gyri, the inferior and superior parietal lobules, the angular gyrus, the middle temporal gyri, and the occipital cortex (Cui et al., 2020; DeWolf et al., 2016; Mock et al., 2018; Sprute, 2013). In contrast, no systematic differences in neural activation have been observed between decimals and natural numbers (DeWolf et al., 2016; Sprute, 2013). Together, these findings suggest that decimal processing is more comparable to natural number processing, aligned with the natural number bias hypothesis, whereas fraction processing relies on a more widely distributed set of brain regions.

Finally, we integrated studies contrasting fraction and decimal processing with nonsymbolic magnitude processing. Overall, these studies have found high overlap between these formats within the IPS in both children and adults (Binzak, 2020; Mock et al., 2018, 2019; Park et al., 2023; Starling Alves, 2021). For instance, Park and colleagues (2023) observed significant neural distance effects within the IPS in response to fractions in fifth-graders, who had been introduced to fractions in schools, but not in second-graders, who had limited exposure to fractions. This same region was associated with neural distance effects in response to nonsymbolic ratio magnitudes in both grades. These findings suggest that regions specialized in nonsymbolic magnitudes may accommodate fractions across development. Aligned with these results, findings from Binzak (2020), Mock and colleagues (2018), and Starling Alves (2021) have corroborated neural distance effects within the IPS in response to both fractions and nonsymbolic ratio magnitudes. These results challenge the lack of grounding hypothesis, which suggests that fractions are not supported by perceptual systems.

#### Multivariate analyses

We then examined how studies investigating rational number processing using a *multivariate* approach (i.e., analyses of activation patterns across multiple voxels within a region) corroborate evidence of overlap and differentiation in neural representations of fractions, decimals, and natural numbers. Importantly, as noted in Table 1, most studies using multivariate approaches have relied on representation similarity analyses. These analyses test the observed pattern of activation against predicted similarity models, indicating whether neural representations across experimental conditions reflect hypothesized relations (Kriegeskorte et al., 2008). Representational similarity analyses are useful for examining if different stimuli, such as fractions and decimals, are represented by similar or distinct neural activation patterns.

For fractions, multivariate analyses have found neural activation maps associated with this format within the IPS and the right prefrontal cortex (PFC) are dissimilar to those associated with natural number processing (DeWolf et al., 2016; Sprute, 2013). Additionally, a multivariate classification analysis showed that fraction processing is associated with greater functional connectivity than natural number processing across several regions, including the left middle temporal gyrus (MTG) and the left inferior parietal lobule, the left inferior frontal gyrus (IFG), the right lingual gyrus, and the right cerebellum (Cui et al., 2020). However, in contrast to these findings, a representational similarity analysis conducted by Bhatia and colleagues (2022) showed that bilateral occipital and middle temporal cortices, in addition to the right IPS, process fractions and natural numbers more similarly, while distinguishing between these symbolic numbers and nonsymbolic magnitudes. Altogether, these results align with findings from univariate analyses, indicating that fraction processing engages more extensive brain regions than natural number processing, even though these formats might still share some common underlying processes.

For decimals, multivariate analyses revealed a more nuanced neural activation pattern than univariate analyses. They suggest that, like fractions, decimal number processing also differs from natural number processing. For example, Sprute (2013) conducted a searchlight classification analysis to investigate the brain regions that distinguished decimals, fractions, and natural numbers. This analysis systematically moves a small "searchlight" window across the brain and uses machine learning to identify patterns of brain activation across experimental conditions (Etzel et al., 2013). Of note, in searchlight analyses, machine learning refers to training a computer algorithm to identify patterns in the data (Etzel et al., 2013), rather than generating new data as in generative artificial intelligence models. Results indicated that the right superior parietal lobule (SPL), the IPS, and the precuneus accurately differentiated between decimals and natural numbers and between fractions and natural numbers (Sprute, 2013). These findings suggest some level of neural specialization for rational numbers within the superior parietal lobule, both in fraction and decimal formats. Importantly, these regions that showed differential processing for fractions and decimals relative to natural numbers are typically associated with numerical magnitude processing and more generalized cognition related to visuospatial processing, working memory, inhibitory control, and conceptual understanding (Binkofski et al., 2016; Caspers et al., 2006; Dadario & Sughrue, 2023; Dormal & Pesenti, 2009).

There is also evidence that decimal number processing differs from fraction and nonsymbolic ratio processing (Mock et al., 2019). Mock and colleagues (2019) presented college students with ratio comparison tasks in several formats: decimal comparison, fraction comparison, pie chart comparison, and comparison of dot proportions (i.e., compare the ratio of yellow to blue dots shown on the left vs. right side of the screen). Using representation similarity analysis, they investigated the similarity of neural representations within the bilateral IPS across these formats. Results, which were comparable across hemispheres, indicated high similarity in neural representation for fractions, pie charts, and dot proportions. In contrast, decimal processing was dissimilar from all these other formats. These findings suggest that fractions and nonsymbolic ratio representations, which feature part–whole ratios, may share underlying mechanisms within the IPS, going in the opposite direction as the lack of grounding hypothesis. In contrast, decimals, which feature base-10 processing, may elicit distinct neural mechanisms.

Altogether, these studies show that the neural mechanisms engaged in rational number processing partially converge with those associated with other formats. In particular, studies show that frontoparietal regions associated with fractions and decimals are also associated with natural numbers and nonsymbolic magnitudes, with the IPS emerging as an important convergence region. Yet multivariate analyses suggest a level of neural specialization for rational numbers, with fractions and decimals being processed dissimilarly to natural numbers.

### ALE meta-analysis

As the majority of the selected studies targeted fraction processing, we conducted an ALE meta-analysis to identify converging brain regions associated with fraction processing. Given the small number of studies targeting decimal processing (i.e., six studies in total with only four reporting target contrasts, two of them being derived from the same sample), the number of independent experiments eligible for inclusion was insufficient to conduct a meaningful ALE meta-analysis (Eickhoff et al., 2016).

In the ALE meta-analysis, we included seven studies (15 contrasts in total) that reported coordinates associated with fraction processing, as identified in univariate analyses. There were 562 combined subjects and 204 foci. We report findings using the minimum cluster size chosen by GingerALE: 704 mm^3^, and results significant at cluster-level family-wise error (FWE) thresholded at *p* <.05, with a voxel-level threshold of *p* <.001 and 1,000 permutations. The dataset with identified coordinates is shared on OSF (https://osf.io/gf254/?view_only=2a30366d355b4486ae74d7e18404bb76).

**Fig. 3:**
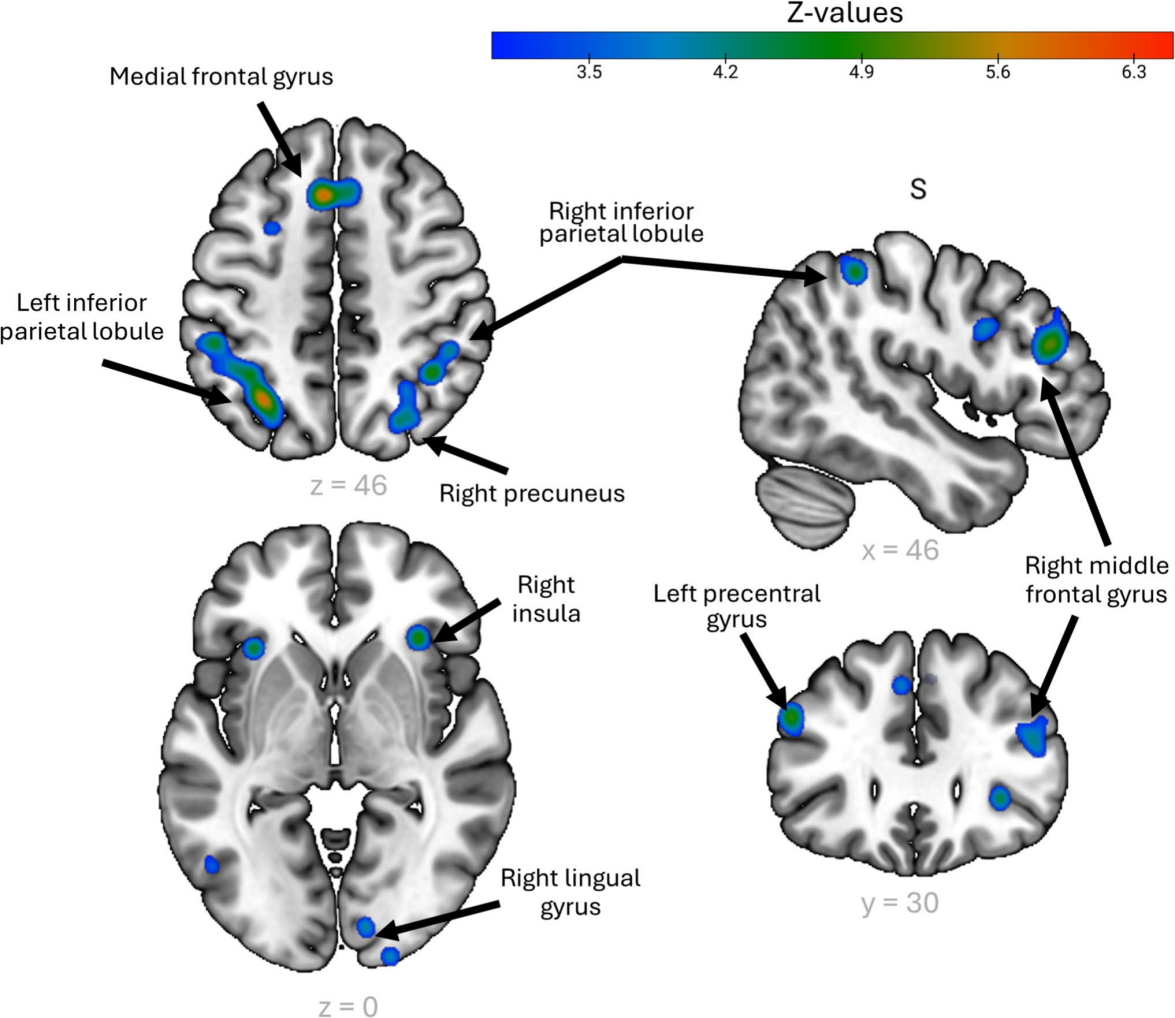
Brain regions associated with fraction processing, as identified by an ALE meta-analysis*.* Spatial distribution of convergent regions for fraction processing according to the ALE meta-analysis. *Z* maps (pFWE <.05) are superimposed on slices from an MNI template (spm152). Overall, clusters around the bilateral inferior parietal lobule, right precuneus, medial frontal gyrus, right middle frontal gyrus, left precentral gyrus, right insula, and right lingual gyrus were identified by the ALE meta-analysis. A leave-one-out sensitivity analysis indicated that the left inferior parietal lobule, right precuneus, left precentral gyrus, medial frontal gyrus, and right middle frontal gyrus were the most robust findings. (Color figure online)

Results (see Fig. 3) revealed eight clusters associated with fraction processing: (1) In the left hemisphere, including the inferior and superior parietal lobules, the intraparietal sulcus, and the precuneus; (2) in the left frontal lobe, centered around the precentral gyrus and encompassing the middle frontal gyrus and a small part of the insula; (3) centered around the medial frontal gyrus, extending bilaterally; (4) centered around the precuneus, also encompassing the right intraparietal sulcus; (5) centered in the middle frontal gyrus; (6) centered around the right inferior parietal lobule; (7) centered in the right occipital lobe, including the lingual gyrus and the inferior occipital gyrus; and (8) around the right insula and the right claustrum. These findings are summarized in Table 2.

**Table 2:** ALE meta-analytic results for functional MRI studies investigating brain regions associated with fraction processing

Cluster	Hemisphere	Region (Brodmann’s area)	Center MNI coordinate			Cluster size (mm^3^)	ALE value	*Z*	Leave one out*
			*x*	*y*	*z*				
1	Left	Inferior parietal lobule (BA 40)	− 33	− 55	46	5,872	0.031	6.21	6/7
2	Left	Precentral gyrus (BA 9)	− 45	12	31	4,448	0.029	5.93	7/7
3	Bilateral	Medial frontal gyrus (BA 6)	− 2	22	46	2,176	0.029	6.00	7/7
4	Right	Precuneus (BA 7)	28	− 65	46	1,944	0.021	4.76	7/7
5	Right	Middle frontal gyrus (BA 9)	46	33	23	1,600	0.024	5.22	7/7
6	Right	Inferior parietal lobule (BA 40)	42	− 45	48	1,312	0.023	5.13	5/7
7	Right	Lingual gyrus (BA 17)	15	− 92	− 3	1,032	0.018	4.32	4/7
8	Right	Insula (BA 13)	33	26	0	712	0.023	5.14	4/7

Cluster-level family-wise error (FWE) thresholded at *p* <.05, with a voxel-level threshold of *p* <.001 and 1,000 permutations. *Indicates in how many leave-one-study-out iterations (max = 7) the cluster was still identified in the ALE meta-analysis. A larger number indicates more robust findings

To interpret findings from the ALE meta-analysis, we used Neurosynth (neurosynth.org). This tool indicated that the regions identified here as linked to fraction processing (Table 2) are not restricted to this skill, but are implicated in neuroimaging meta-analyses as being associated with many other cognitive functions (see list in Supplementary Materials, Table S2). For instance, the inferior parietal lobule has been associated with natural number processing, working memory, visuospatial processing, and attention mechanisms. The precentral gyrus, the middle frontal gyrus have been associated with working memory, cognitive control, and semantic understanding. The medial frontal gyrus has been associated with working memory and cognitive control. Altogether, the regions identified in the ALE meta-analyses are connected with cognitive functions relevant to multiple theoretical accounts of fraction processing, including the natural number bias hypothesis (i.e., natural number processing and cognitive control) and the cognitive load hypothesis (i.e., working memory). These findings suggest that fraction processing likely engages multiple cognitive systems, consistent with the possibility that these theoretical accounts are not mutually exclusive mechanisms, but rather operate together in fraction processing. Importantly, the overlap between brain regions associated with nonsymbolic magnitude processing and fraction processing challenges the view that fractions lack grounding in perceptual magnitude systems.

We then followed up with a sensitivity analysis. We reran the ALE meta-analysis by iteratively leaving each study out to check how much it contributed to the main findings (Leroy et al., 2020; Wilson et al., 2018). The left precentral gyrus, medial frontal gyrus, right precuneus, middle frontal gyrus, and left inferior parietal lobule emerged as the most robust regions associated with fraction processing in this analysis, consistently converging across all leave-one-out iterations. In contrast, the right inferior parietal lobule, right lingual gyrus, and right insula did not survive all iterations of the sensitivity analyses. This pattern suggests that convergence in these regions may be more sensitive to the inclusion of individual studies, rather than reflecting consistent convergence across the full set of experiments. While these areas may also be associated with fraction processing, the evidence supporting their involvement is comparatively less robust and should be interpreted with greater caution. We also conducted two exploratory analyses by testing (1) only neural distance effects linked to fractions and (2) only fractions versus natural numbers contrasts. As shown in Supplementary Materials Tables S3 and S4, the converging areas highly overlapped with areas identified in the main analysis.

It is important to note that both the ALE and the sensitivity analyses were underpowered. For instance, some studies suggest that ALE meta-analysis yields more reliable results when including around 17 studies (Eickhoff et al., 2016; Müller et al., 2018). A smaller number of studies can lead to disproportionate influence of individual experiments, with single studies contributing to a large part of convergence. While the sensitivity analysis is a good alternative, examining the stability of results when each study is removed, their ability to detect smaller or more spatially variable effects is also limited when the overall number of studies is small. Thus, some regions associated with fraction knowledge may not have been identified in the results. These limitations highlight the need to interpret results with caution. As the number of studies using neuroimaging to investigate fraction processing increases, future meta-analytic procedures with more observations should be conducted to establish more precise estimates of the brain regions linked to fraction processing.

## Discussion

Rational number knowledge predicts important academic outcomes, being considered a gatekeeper skill (Booth et al., 2014; Wu, 2001). Despite its importance, mastery of rational numbers is recognized as challenging, with systematic misconceptions and errors in arithmetic problems being observed throughout development (e.g., Gabriel et al., 2013; Schneider & Siegler, 2010). There is still an ongoing debate regarding the cognitive factors underlying these difficulties. To date, three main accounts have been proposed: (1) The lack of grounding hypothesis, which argues that rational number difficulties are related to the absence of perceptual systems that directly support their representation (Feigenson et al., 2004); (2) the cognitive load hypothesis, which attributes these difficulties to the high demands rational numbers place on attentional and executive control systems (Lortie-Forgues et al., 2015); and (3) the natural number bias hypothesis, which argues that rational number difficulties emerge due to interference from prior natural number knowledge (Ni & Zhou, 2005). Identifying mechanisms associated with rational number processing can inform these theories and contribute to broader cognitive and educational frameworks for rational number understanding.

In this systematic review, we targeted neural mechanisms linked to rational number knowledge. In particular, we integrated fMRI research investigating the brain regions associated with fraction and decimal processing. Overall, the studies suggest that fractions and decimals engage frontoparietal regions with the intraparietal sulcus playing a central role. Importantly, the brain regions linked to rational number processing (and particularly those identified for fractions using Neurosynth) are also implicated in nonsymbolic magnitude processing, natural number processing, and executive functions, which have implications for the grounding, the cognitive load, and the natural number bias hypotheses. These results suggest that cognitive hypotheses for rational number processing may not be mutually exclusive. At the same time, despite overlapping activation patterns, fractions and decimals also showed distinct neural representations both relative to each other and to other numerical formats, as demonstrated by MVPA findings. These results suggest that rational number processing may involve both shared and format-specific neural mechanisms.

In the following paragraphs, we discuss findings related to fraction processing and decimal processing, and connect them with theoretical accounts of rational number processing. We conclude by pointing out limitations and outlining directions for future research.

### Brain regions associated with fraction processing

The majority of the studies selected in this review investigated fraction processing. When conducting a summary of these studies, we found that fraction processing is associated with fronto-parietal brain regions, in particular the intraparietal sulcus (Bhatia et al., 2022; DeWolf et al., 2016; Ischebeck et al., 2009; Park et al., 2023). These regions have also been identified as relevant for fraction processing in previous reviews (Obersteiner et al., 2019; Rosenberg-Lee, 2021). The IPS, in particular, has been identified as a hub for magnitude processing (Bugden et al., 2012; Dormal & Pesenti, 2009), being implicated in natural number processing (Holloway & Ansari, 2009; Piazza et al., 2007; Vogel et al., 2017), decimal processing (DeWolf et al., 2016; Sprute, 2013), and nonsymbolic magnitude processing (Jacob & Nieder, 2009; Mock et al., 2018, 2019; Piazza et al., 2004).

Although frontoparietal regions associated with fraction processing are also recruited during other numerical tasks, significant contrasts between fractions and other number formats indicate distinct levels and patterns of neural activation. For instance, the descriptive summary of both univariate analyses and MVPA converged in showing that fraction processing differs from decimal number processing and natural number processing within a range of regions including the intraparietal sulcus, the middle frontal gyrus, and the middle temporal gyrus (Cui et al., 2020; DeWolf et al., 2016; Mock et al., 2018, 2019; Sprute, 2013). These differences suggest format-dependent neural responses, which may reflect differences in underlying cognitive mechanisms.

To quantitatively integrate and identify consistent neural correlates across studies, we conducted an ALE meta-analysis of fraction processing. The results further showed convergent activation for fraction processing in the bilateral inferior parietal lobule, the bilateral intraparietal sulcus, the precentral gyrus, the middle frontal gyrus, and the right precuneus. To characterize the broader functional profile of these regions, we examined their associated terms in Neurosynth (see Supplementary Materials, Table S2). The brain regions we identified as associated with fraction processing were also linked to other cognitive functions, such as working memory and cognitive control (Duncan & Owen, 2000; Fedorenko et al., 2013). Regions associated with working memory being also engaged in fraction processing can be interpreted as evidence that fractions are cognitively demanding, which is aligned with the cognitive load hypothesis. In addition, the engagement of systems implied in cognitive control in fraction processing aligns with predictors made by the natural number bias hypothesis. It is possible that, when processing fractions, people rely on prior natural number knowledge, requiring cognitive control to inhibit these representations.

The inferior parietal lobule, encompassing the intraparietal sulcus (IPS), emerged as one of the most robust clusters in the ALE meta-analysis. Although nonsymbolic magnitude processing was not among the most frequently associated terms for this region in Neurosynth, converging evidence has linked activity within the IPS to nonsymbolic magnitude processing (Houdé et al., 2010; Kaufmann et al., 2011; Sokolowski et al., 2021). Consistent with this broader literature, univariate and MVPA studies qualitatively reviewed here have reported that fractions and nonsymbolic ratio processing share neural correlates (e.g., Mock et al., 2018, 2019; Park et al., 2023). Together, these findings diverge from predictions that rational number processing is challenging due to a lack of perceptual systems that grounds it. Instead, they suggest that fractions may be supported, at least in part, by neural systems involved in processing perceptually based magnitudes.

In summary, the description of fMRI studies targeting fraction processing, along with an ALE meta-analysis, indicates that fractions are associated with activity within the IPS and the precuneus, bilaterally, along with the left precentral gyrus, the right middle frontal gyrus, and bilateral medial frontal gyrus. These findings are consistent with interpretations that fraction processing engages cognitive control and working memory systems, aligning with the natural number bias and the cognitive load hypotheses. However, they do not offer strong support for the hypothesis that fractions lack related perceptual systems.

### Brain regions associated with decimal processing

Despite using broad search terms across six databases and including theses, dissertations, and preprints in our review, we identified only six fMRI studies investigating decimal number processing. This limited number is surprising, given that decimal numbers are frequently used to convey everyday information—such as prices, speed, distances, and weight—yet still pose challenges for many people (D’Ambrosio & Kastberg, 2012; Durkin & Rittle-Johnson, 2015; Lortie-Forgues et al., 2015). As some of these studies did not report detailed contrasts for decimal processing, we restricted our analyses of decimals to an integrative summary of findings, not relying on ALE procedures.

Overall, univariate analyses suggest that decimal number processing is associated with the bilateral intraparietal sulcus, left fusiform gyrus, left frontal gyrus, and left insula (DeWolf et al., 2016; Mock et al., 2018, 2019; Sprute, 2013). These regions have been reported by prior studies as also linked to natural number processing (Mock et al., 2018; Sokolowski et al., 2021), which raises the possibility that these numerical representations share underlying cognitive mechanisms. Aligned with this interpretation, contrasts between neural activation elicited by decimals and natural numbers have revealed no significant differences (e.g., DeWolf et al., 2016).

Behavioral evidence further supports the possibility that decimal processing engages mechanisms also involved in natural number processing. For example, an eye-tracking and computational model study demonstrated that decimals with an unequal number of decimal places (i.e., different length, such as 7.14 vs. 7.6) lead to systematic errors compatible with application of a natural number property whereby a larger number of digits indicate larger magnitudes (e.g., 714 > 76; Huber et al., 2014). Behavioral studies also indicate that, when comparing decimal magnitudes, participants often ignore the decimal point and apply natural number heuristics (DeWolf et al., 2014). Because the structure of decimals is closely aligned with the base-10 system, their processing may recruit the same mechanisms engaged in natural number processing. This interpretation aligns with the natural number bias hypothesis (Ni & Zhou, 2005), which proposes that prior expertise with natural numbers influences reasoning about rational numbers.

Findings from Mock and colleagues (2019) further support the hypothesis that decimals engage neural representations linked to base-10 symbolic structure, which they argue is shared with natural numbers. Using representation similarity analyses, they found that activation patterns associated with processing part–whole relations, as represented by fractions and nonsymbolic ratio magnitudes, were highly similar between each other. In contrast, decimal processing was highly dissimilar from these other formats. Because decimals have a base-10 structure, which is also a defining feature of natural numbers, these findings suggest that base-10 symbolic formats engage neural representations that differ from those supporting magnitudes based on part–whole relations. Although natural numbers were not directly examined in this study, this interpretation aligns with the proposal that decimals and natural numbers recruit similar neural mechanisms, corroborating the natural number bias hypothesis (Ni & Zhou, 2005).

In contrast with these results, other evidence indicates that decimal processing may not fully overlap with other numbers with base-10 structure, like natural numbers. In a searchlight study, the precuneus, lingual gyrus, occipital gyrus, and temporal gyrus reliably differentiated decimals from natural numbers (Sprute, 2013). Prior studies have linked these regions to language, visual processing, and cognitive control (Dadario & Sughrue, 2023; Davey et al., 2016; Palejwala et al., 2021), functions that may contribute to the unique processing demands of decimals relative to natural numbers. Some behavioral studies support this interpretation. For instance, Hurst and Cordes (2016) found that children were slower and showed greater ratio effect when comparing two decimals than when comparing two natural numbers. Moreover, children showed high performance (> 92% accurate) when comparing decimals versus natural numbers, even when experimental manipulations could lead to the natural number bias (e.g., 5 vs. 2.10, where ignoring the decimal point would lead to the wrong response). These results suggest that children may engage distinct strategies when processing decimals and natural numbers, such that the natural number bias may not fully account for decimal processing.

Factors underlying the divergent findings relative to decimal processing remain unclear. While some of these findings suggest that decimals may rely on base-10 representations consistent with natural number processing, other MVPA studies directly comparing decimals and natural numbers have found dissimilar neural representations. One possibility is that MVPA can detect differences between decimals and natural numbers because these distinctions emerge only when distributed activation patterns across voxels are considered.

Alternative explanations relate to differences in the stimuli lists across studies. For example, DeWolf and colleagues (2016) presented participants only with decimals to the hundredths place (e.g.,.89), whereas Sprute (2013) included decimals to both the tenths (e.g.,.4) and hundredths place (e.g.,.89). Behavioral studies have shown that comparing decimals with different number of decimal places can lead to errors if participants rely on natural number heuristics (e.g., indicating that.5 is smaller than.29 because 5 > 29; Rosenberg-Lee et al., 2023; Varma & Karl, 2013). Thus, due to the inclusion of decimals with mixed place values in Sprute’s (2013) paradigm, participants may have engaged in strategies that recruit different brain regions relative to those associated with natural numbers. This possible strategy shift elicited by the stimuli list could account for the distinct neural activation patterns observed across decimal studies. Therefore, a clearer understanding of the similarities and differences between decimal and natural number processing requires further studies that explicitly account for stimulus characteristics, such as the length of the decimal numbers.

In summary, the description of fMRI studies targeting decimal processing indicates that it is associated with activity in the bilateral intraparietal sulcus, left fusiform gyrus, left frontal gyrus, and left insula. Several studies suggest common brain regions engaged during decimal and natural number processing, which is consistent with the natural number bias hypothesis (Ni & Zhou, 2005). In contrast, findings from MVPA studies indicate differences in neural representational patterns for decimals and natural numbers, suggesting that they may also engage distinct mechanisms. Additional evidence is needed to clarify how these mechanisms relate to theoretical frameworks such as the lack of grounding hypothesis and the cognitive load hypothesis.

### Cognitive accounts for rational number processing

As discussed above, neuroimaging studies investigating fraction and decimal number processing indicate that these skills are associated with brain regions also implicated in nonsymbolic magnitude processing, higher-order cognition, and natural number processing. On the one hand, these findings suggest that the natural number bias and the cognitive load hypotheses are plausible explanations for challenges observed in rational number processing. On the other hand, they provide lower support for the lack of grounding hypothesis. Although neuroimaging evidence alone is insufficient to resolve the debate among these theoretical approaches, it remains informative, particularly when considered alongside behavioral findings.

Diverging from the lack of grounding hypothesis, results have shown that rational number processing, particularly fraction processing, recruits brain regions also associated with nonsymbolic magnitude processing. Aligned with these findings, correlational and experimental behavioral studies have shown that nonsymbolic magnitude processing is associated with fraction skills (Gouet et al., 2020; Hansen et al., 2015; Matthews et al., 2016). Consistent with the possibility that rational numbers are grounded in perceptually based magnitude systems, Lewis and colleagues (2016) have proposed that humans are equipped with a neurocognitive architecture specialized in processing nonsymbolic ratios: the ratio processing system (RPS). The RPS account suggests that this system, linked to activity within the IPS, develops very early in humans and has a phylogenetic basis. Across development, it may support the understanding of symbolic ratio magnitudes. Under this perspective, difficulties with rational number understanding may emerge due to poor leverage of this system, contextual factors such as pedagogical practices that rely largely on absolute magnitudes and natural numbers when teaching symbolic rational numbers, or increased cognitive load (Lewis et al., 2016).

Converging behavioral evidence supports the involvement of higher-order cognition in fraction and decimal number understanding, indicating high cognitive load due to processing demands or engagements of inhibitory mechanisms. For instance, people who perform better in inhibitory control and relational reasoning tasks also tend to perform better in fraction and decimal tasks (Avgerinou & Tolmie, 2020; Kalra et al., 2020; Leib et al., 2023; Park & Matthews, 2025; Ye et al., 2016). As posited by the natural number bias hypothesis, these skills may support fraction understanding by suppressing inappropriate natural number heuristics and supporting adoption of more efficient processing strategies (Ni & Zhou, 2005). Corroborating this hypothesis, Miller Singley and Bunge (2018) showed that people tend to apply componential strategies that reflect natural number bias when reasoning about fraction magnitudes (i.e., focusing on numerators or denominators alone), but shift to holistic strategies (i.e., focusing of the absolute fraction magnitude) when the componential approaches are less effective. In particular, rather than explicitly calculating magnitudes (e.g., dividing numerator by denominator), participants often leverage relational reasoning to compare fractions.

Variation in strategies adopted to process fractions and decimals can also lead to increased demands in working memory (Hurst & Cordes, 2017), which also provides support for the cognitive load hypothesis. Importantly, higher working memory capacity has been associated with better performance in fraction and decimal tasks (Coulanges et al., 2021; Leib et al., 2023; Seethaler et al., 2011). Thus, the engagement of higher order cognition at the behavioral level, aligned with activation of frontal regions in response to rational number tasks, can be interpreted as evidence for both the natural number bias hypothesis and the cognitive load hypothesis, suggesting these hypotheses are not mutually exclusive.

Together, these findings suggest that rational number processing relies on interactions between magnitude representations and higher-order cognitive systems, supporting accounts that emphasize both grounding in perceptual magnitude and the role of executive functions. However, important nuances should be considered. For instance, it remains unclear how decimal numbers relate to nonsymbolic magnitude representations, making it challenging to assess the role of the lack of grounding hypothesis in decimal processing. Additionally, some findings suggest greater neural mechanisms shared between decimals and natural numbers than between fractions and natural numbers. Thus, it is not fully clear if sources of natural number bias are fully shared across fractions and decimals. These considerations raise the possibility that theoretical accounts of rational number difficulties may be, at least in part, format-dependent. Future investigations, integrating both behavioral and neuroimaging approaches, are needed to clarify how different numerical formats relate to magnitude representations and higher-order cognitive mechanisms.

### Limitations and future directions for the field

The reviewed studies leave several open questions about the brain regions, as well as the cognitive mechanisms, associated with fraction and decimal processing, highlighting important avenues for future research. First, the number of studies in this area remains limited, which requires caution when interpreting our findings. In particular, our ALE meta-analysis was underpowered (Müller et al., 2018). Although the leave-one-out sensitivity analysis (Leroy et al., 2020; Wilson et al., 2018) suggested that the overall ALE meta-analysis findings were not driven by a single study, the converging brain regions identified for fraction processing should be viewed as an exploratory result in need of replication as the number of studies in this area continues to grow.

Since the date of our search for studies and extraction of their findings, new neuroimaging studies targeting fraction and decimal processing have been published. For instance, using representation similarity analysis, Abreu-Mendoza and colleagues (2026) found that IPS activation for fractions was more similar to discretized nonsymbolic ratio magnitudes (e.g., stacked bars with partitions) than continuous nonsymbolic ratio magnitudes (e.g., stacked bars without partitions), in particular when inhibitory control was needed. In addition, using decimal comparisons with a varied number of decimal places, Rosenberg-Lee and colleagues (2026) found evidence for the natural number bias within the IPS and the insula. In contrast, processing of the rational magnitudes represented by decimals was associated with regions linked to cognitive control, such as the anterior cingulate cortex. These two studies converge with findings from the present review by showing that rational number processing relates to both magnitude processing and inhibitory control mechanisms. Despite their important contributions, more studies are still needed to better disentangle the role of these cognitive functions on rational number processing. One important direction, in particular, is to expand research on mechanisms linked to decimal processing, as most studies have focused on fractions. As discussed by Rosenberg-Lee (2021), number formats that share a base-10 structure with whole numbers, like decimals, offer a compelling case to study the interference of natural numbers in processing rational numbers, requiring further investigations.

Another limitation relates to the robustness of the evidence. In this study, we adopted a more lenient approach by including as many studies as possible, given the limited number of fMRI investigations on rational number knowledge. However, methodological limitations in some of the included studies may impact the reliability of their findings. There is an ongoing debate about how methodological aspects of fMRI studies—such as sample size, the amount of individual-level data, and experimental design—influence replicability (Bennett & Miller, 2013; Larsson et al., 2016; Nee, 2019; Yarkoni, 2009). The majority of studies selected in this review conducted group analyses with small sample sizes (all *n* < 70, 11 studies with *n* < 30), meaning they might be underpowered (Mumford & Nichols, 2008; Yarkoni, 2009). Moreover, most studies relied on comparison paradigms, with only a few using different experimental designs, such as adaptation tasks. Due to this methodological choice, it is difficult to determine how much the brain regions identified in this review as associated with fraction and decimal processing are actually a product of explicit comparison experimental paradigms, such as the increased attention, working memory, and response selection demands of the active and relatively difficult task of comparing fractions.

Beyond task demands that may confound results, stimulus features may also influence the current findings. For example, fractions involve two main visual components per fraction (numerator and denominator divided by a line) that, in relation to each other, form one holistic magnitude. Single digits, multi-digit numbers, and decimals all have place value that gives each number representational meaning without reference to the other numbers. Fractions, on the other hand, require the viewer to consider the relative magnitudes of each number simultaneously. These stimulus features may influence the results of any task that requires participants to actively compare fraction magnitudes such that they also index generalized task-processing nonspecific to fractions, and visual processing that may be common to other types of symbols or relational comparisons. That said, increased difficulty and relational thinking are inherent aspects of rational number processing that cannot be disambiguated from thinking about their magnitudes. Future studies should investigate rational number processing using a broader range of experimental paradigms such as active vs. passive paradigms, or nuanced parametric modulations with stimuli that are tightly controlled for visual features to determine the extent to which task design has influenced current findings.

The robustness of the evidence may also be influenced by analytical procedures. While some of the selected studies used multivoxel pattern analysis (MVPA), univariate analyses have remained the norm. Research has shown that MVPA can complement univariate approaches by capturing distributed patterns of neural activation, rather than focusing only on statistical inference at the voxel level (Haxby, 2012; Norman et al., 2006). While univariate analyses can identify brain regions associated with fraction and decimal processing and help localize relevant coordinates, MVPA may be more sensitive to nuanced patterns of neural activation that distinguish these representations from each other and from other numerical formats.

In fact, MVPA studies included in this review have detected significant differences between fraction and decimal processing and other number formats (e.g., natural numbers, nonsymbolic Magnitudes; Bathia et al., 2022; DeWolf et al., 2016; Sprute, 2013). Examining neural mechanisms shared across fractions, decimals, natural numbers, nonsymbolic magnitudes, and higher-order cognition is an important step toward informing theoretical accounts of rational number processing. Future fMRI studies should incorporate MVPA to better characterize format-dependent neural activation patterns, testing the lack of grounding, cognitive load, and natural number bias hypotheses. Furthermore, because fMRI findings are correlational in nature (Brown et al., 2007; Logothetis, 2008), their interpretation should be complemented by evidence from other experimental approaches. These include intervention studies, longitudinal designs, neuropsychological investigations, and other neuroimaging methods such as transcranial magnetic stimulation. Relying on psychometrically tested tools, these approaches can help clarify how different cognitive functions are associated with rational number processing.

Developmental aspects of rational number knowledge and the brain systems involved are also important avenues for future research. Most of the reviewed studies focused on adult populations, with the brain regions associated with fraction and decimal processing across developmental stages remaining unexplored. Among the studies reviewed, only two—conducted by the same research group—focused on children (Park et al., 2023; Starling Alves, 2021). These studies provided evidence that brain regions linked to nonsymbolic ratio magnitude processing may also be associated with fraction processing. However, they did not examine how this processing relates to other number formats, such as decimal and natural number processing, or to broader cognitive functions. Thus, it remains unclear which neural mechanisms are associated with fractions and decimals from early in development.

For instance, it is possible that these numbers initially rely on the same brain regions that start diverging with maturation and education, similar to the symbolic estrangement theory that has been proposed for the developmental association between symbolic and nonsymbolic numbers (Lyons et al., 2012). Future studies should target different rational numbers in children to fully understand the brain regions associated with processing these magnitudes across development. Developmental studies could not only inform the academic community on how maturation and education influence these processes, but also uncover how different brain regions are specifically associated with either fractions or decimals, or overlap in processing both of them. Moreover, studies targeting functional connectivity can highlight how brain regions support fractions and decimals in a coordinated way, moving from a focus on isolated brain regions toward understanding network interactions. These studies can provide information on neural mechanisms underlying shared and specific fraction and decimal processing, which may inform developmental accounts of how these skills emerge and refine.

How individual differences influence fraction and decimal processing—and the findings from fMRI studies—is also an important open question. Behavioral studies have shown that several cognitive skills, such as inhibitory control, language, and relational reasoning, influence performance on fraction and decimal tasks (Avgerinou & Tolmie, 2020; Coulanges et al., 2021; Gómez et al., 2015; Kalra et al., 2020; Leib et al., 2023; Park & Matthews, 2025). Depending on a dynamic interaction between their cognitive abilities, prior knowledge, and task-specific features, individuals may engage in different strategies when processing fractions and decimals (Alibali & Sidney, 2015; Braithwaite & Rafferty, 2025). For instance, decimal comparison tasks in which all numbers have the same number of decimal places may elicit different response patterns than tasks with numbers that vary in decimal length (Huber et al., 2014; Rosenberg-Lee et al., 2023; Varma & Karl, 2013). Similarly, features of the stimuli in fraction comparison tasks, such as shared numerators or denominators or proximity to a benchmark like ½, can influence processing strategies and outcomes (Morales et al., 2020; Obersteiner et al., 2022; Toomarian & Hubbard, 2018).

Although some fMRI findings suggest that individual differences and strategy use may influence the brain regions recruited for decimal and fraction processing (Ischebeck et al., 2009; Sprute, 2013), further investigation is needed to clarify these effects. Future studies should disentangle the processing mechanisms dedicated to magnitude representation of rational numbers from the various strategies used to compare and calculate with rational numbers. Strategies can be directly accounted for by employing strategy reports taken outside of the scanner or by inferring strategy through the use of eye-tracking data (Huber et al., 2014; Obersteiner & Tumpek, 2016; Plummer et al., 2017), or by employing more nuanced analyses of behavioral data with evidence-accumulation models such as drift diffusion models (Myers et al., 2022; Ratcliff, 2022). Analyzing this data in concert with carefully controlled experimental paradigms will provide a more robust account of what neural mechanisms are being employed for what purpose in the context of fraction and decimal magnitude processing.

The interaction of strategy use with prior knowledge should also be considered for studies of children with math learning difficulties. Children with math difficulties start middle school behind their peers in rational number understanding and continue to fall further behind during this critical transition to algebra (Lortie-Forgues et al., 2015; Mazzocco et al., 2013; Tian & Siegler, 2017). Therefore, understanding what the various impediments are to early fraction and decimal understanding, along with underlying cognitive mechanisms, will be critical for improving educational outcomes for at-risk groups.

## Conclusion

This systematic review investigated the brain regions associated with fraction and decimal number processing. The reviewed fMRI studies demonstrated that processing these rational numbers is associated with frontoparietal brain regions, with an important role of the intraparietal sulcus. These regions are also linked to nonsymbolic magnitude processing, natural number processing, and higher-order cognition, providing support for theoretical accounts arguing that rational numbers rely on perceptual systems, but are cognitively demanding and prone to interference from prior natural number knowledge. However, these findings should be interpreted with caution given the limited number of available studies. Additionally, brain regions identified in this review might have been highly influenced by methodological factors such as stimuli selection, which warrants further investigations. As neuroimaging research continues to grow, future studies investigating the neural basis of fraction and decimal processing are still needed to disentangle different theoretical accounts for rational number processing.

## Supplementary Information

Below is the link to the electronic supplementary material.

Supplementary file1 (DOCX 404 kb)

## Data Availability

The search terms and protocol for the systematic review have been made available on the OSF (https://osf.io/gf254/?view_only=2a30366d355b4486ae74d7e18404bb76). No original data were used in preparing the current manuscript.

## Code availability

NA.
