# Supplementary material for: Rational numbers: A systematic review and ALE meta-analysis of the neuroimaging of fraction and decimal processing in the brain
Source: Psychon Bull Rev. 2026 Jun 15;33(5):174. doi: 10.3758/s13423-026-02939-y (PMC13269167; doi:10.3758/s13423-026-02939-y)
Supplement: Supplementary file 1 — Supplementary file1 (DOCX 404 kb) [file 13423_2026_2939_MOESM1_ESM.docx]

**Rational Numbers: A Systematic Review and ALE Meta-Analysis of the Neuroimaging of Fraction and Decimal Processing in the Brain**

**Supplementary Materials.**

Table S1. Summary of studies methods and main results

Table S2. Most commonly associated processing-related terms for each coordinate from neurosynth.org for each meta-analytic cluster.

Table S3. Sensitivity analysis: ALE meta-analytic results for functional MRI studies investigating fraction near distance vs. fraction far distance.

Table S4. Sensitivity analysis: ALE meta-analytic results for functional MRI studies investigating fraction vs. natural number contrasts.

**Table S1. Summary of studies methods and main results**

| **Reference** | **Neuroimaging Method** | **Contrasts** | **Main Results** |
| --- | --- | --- | --- |
| Bhatia et al., 2022 | fMRI, univariate analysis and MVPA | - Adapted vs. deviant magnitudes - Whole number vs. fractions | **Univariate analyses:**  Adapted vs. deviant magnitudes:   - Adaptation effect for whole numbers in the left fusiform gyrus, but no effect for fractions   **MVPA:**  Representational Similarity Analysis (RSA):   - Absolute vs. relative magnitudes: right occipital cortex and left rostro-lateral prefrontal cortex - Lines vs. numbers: bilateral occipital and middle temporal cortices, and right intraparietal sulcus (IPS) |
| Binzak, 2020 | fMRI, univariate analysis | - Near vs. far distances - Nonsymbolic vs. symbolic vs. mixed (nonsymbolic vs. symbolic) condition | **Univariate analyses:**  Near vs. far:   - intraparietal sulcus - bilateral frontal lobe - anterior insula - lateral and inferior occipital lobe - left fusiform gyrus - premotor area   Symbolic vs. nonsymbolic:   - right anterior insula - left inferior frontal gyrus - left fusiform gyrus - left intraparietal sulcus - supplementary motor area   Nonsymbolic vs. symbolic:   - right inferior parietal lobule - right visual association area - parahippocampus - superior occipital gyrus   Mixed vs. nonsymbolic + symbolic:   - bilateral fusiform gyrus - left intraparietal sulcus - right inferior frontal gyrus - bilateral primary motor cortex   Conjunction nonsymbolic and symbolic:   - left and right superior parietal lobule - right anterior insula - right inferior frontal gyrus - left ventrolateral prefrontal cortex - supplementary motor area |
| Cui et al., 2020 | fMRI, univariate analysis | - Near vs. far distance - Whole number vs. fractions | **Univariate analyses:**  Near vs. far distance:   - bilateral superior and inferior parietal lobules, occipital cortex, and middle frontal gyrus - Greater functional connectivity within the left middle temporal gyrus (MTG) and from this region to the left middle frontal gyrus (IFG) in near vs. far distance fractions - No significant functional connectivity effects for near vs far distance whole numbers   Whole number vs. fractions:   - Bilateral superior and inferior parietal lobules, occipital lobules, middle frontal gyrus, and left middle and inferior temporal gyrus - Greater functional connectivity from left MTG to left inferior parietal lobule, left IFG, right lingual gyrus, and right cerebellum for near distance fractions than near distance whole numbers - Greater functional connectivity from left MTG to left precentral gyrus, left superior frontal gyrus, and left superior parietal lobule for far distance fractions than far distance whole numbers - Functional connectivity from left MTG to left middle temporal gyrus and left middle frontal gyrus were greater when comparing short-distance fractions than when comparing long-distance fractions. - No brain regions survived the significance test for the difference of connectivity intensity in the short-distance whole numbers vs. long-distance whole numbers contrast.   **MVPA:**  Classification analysis:   - Difference in fraction vs. natural number processing within the bilateral superior and inferior parietal lobules, occipital lobules, middle frontal gyrus, and left middle and inferior temporal gyrus |
| DeWolf et al., 2016 | fMRI, univariate analysis and MVPA | - Near vs. far distance - Whole number vs. fractions vs. decimals | **Univariate analyses:**  Fractions vs. decimals:   - Left intraparietal sulcus (IPS), junction of the intraparietal and intraoccipital sulci, precentral gyrus, superior and middle frontal gyri, and in temporal cortex, inferior and middle temporal gyri, and right cerebellum   Fractions vs. whole numbers:   - Bilateral IPS, precentral gyrus, superior and middle frontal gyri, inferior and middle temporal gyri, cerebellum   Decimals vs. whole numbers:   - n.s.   **MVPA:**  RSA:  Fraction vs. Decimal model:   - Left IPS   Fraction vs. whole-number model:   - Bilateral IPS   Decimal vs. whole number model:   - Bilateral IPS, but fewer voxels |
| Fassbender et al., 2014 | fMRI, univariate analysis | - Round vs. decimal value in financial context (e.g., R$25.00 vs. R$25.12) | **Univariate analyses:**  Round vs. decimal value   - Stronger activity in the nucleus accumbens for round than decimal values |
| Ischebeck et al., 2009 | fMRI, univariate analysis | - Near vs. far distance - Fractions with same numerator vs. same denominator vs. congruent vs. incongruent conditions | **Univariate analyses:**  Same numerator vs. same denominator:   - Middle and inferior frontal areas, intraparietal sulcus (IPS), supplementary motor area (SMA), and occipital lobe   Congruent vs. same numerator:   - Right inferior frontal gyrus (IFG), medial frontal gyrus   Incongruent vs. congruent   - Left inferior and medial frontal gyrus, SMA, cingulate cortex   Near vs. far distance between fraction components:   - n.s. effect   Near vs. far holistic distance:   - Right IPS and middle occipital gyrus   Conjunction analyses for conditions:   - Bilateral superior parietal lobule, insula, rIFG, supramarginal gyrus |
| Jacob & Nieder, 2009 | fMRI, univariate analysis | - Near vs. far distance - Adapted vs. deviant ratios | **Univariate analyses:**  Near vs. far:   - Intraparietal sulcus (IPS) and, anterior and middle cingulate cortex, and left precentral cortex for Arabic digits and words   Deviant vs. adapted:   - Anterior IPS and superior parietal lobule |
| Mock et al., 2018 | fMRI, univariate analysis | - Near vs. far distance - Nonsymbolic vs. symbolic ratios - Fractions vs. decimals vs. dot ratios vs. pie ratios | **Univariate analyses:**  Near vs. far:   - Fraction: right intraparietal sulcus (IPS), bilateral supplementary motor area (SMA), and bilateral frontal gyrus - Decimals: bilateral IPS, left occipito-temporal regions, left fusiform gyrus, left frontal gyrus, left insula, and bilateral precentral gyrus - Pie chart: bilateral IPS, bilateral occipital regions, bilateral inferior frontal gyrus (IFG), bilateral insula, bilateral precentral gyrus, and bilateral midcingulate cortex (MCC) - Dot ratios: bilateral IPS, left anterior cingulate cortex, right superior frontal gyrus (SFG), and inferior occipital gyrus   Symbolic vs. nonsymbolic:   - bilateral middle frontal gyrus, left SFG, right SMA and left angular gyrus (AG)   Nonsymbolic vs. symbolic:   - parietal and occipital cortex, left middle occipital gyrus, right MCC, insula and SFG   Conjunction nonsymbolic + symbolic:   - right superior parietal lobule (hIP3) as well as bilateral occipital regions |
| Mock et al., 2019 | fMRI, univariate analysis and MVPA | - Near vs. far distance - Nonsymbolic vs. symbolic ratios - Fractions vs. decimals vs. dot ratios vs. pie ratios | **Univariate analyses:**  Fractions + dot ratios + pie ratios vs. decimals :   - Bilateral inferior parietal lobule, middle frontal gyrus, bilateral supplementary motor area (SMA), and bilateral insula   Conjunction analysis:   - Visual occipital areas, bilateral thalamus, supplementary motor areas, inferior parietal cortex   **MVPA:**  RSA:  Left and right hemispheres:   - Dot ratio similar to fractions and pie ratios - Decimals different from dot ratios - Pie ratios more similar to decimals - Decimals different from fractions |
| Park et al., 2025 | fMRI, univariate analysis and MVPA | - Near vs. far distances - Nonsymbolic vs. symbolic vs. mixed (nonsymbolic vs. symbolic) condition - 2nd vs. 5th graders | **Univariate analyses:**  Near vs. Far:  2^nd^ grade:   - Overall: Bilateral superior parietal lobules, intraparietal sulcus (IPS), middle frontal gyrus, and anterior insula - Nonsymbolic: bilateral parietal lobules and right inferior frontal gyrus - Mixed: broader frontal and parietal regions, inferior frontal gyrus (IFG), frontal pole - Fractions: ns   5^th^ grade:   - Bilateral superior parietal lobules, IPS, middle frontal gyrus, insula, frontal regions - Nonsymbolic: parietal and frontal regions - Mixed: parietal and frontal regions - Fractions: parietal and frontal regions   Conjunction analyses:  5^th^ graders:   - IPS, frontal pole   2^nd^ vs. 5^th^ grade   - Lateral occipital cortex, IPS, superior parietal lobule, posterior middle temporal gyrus, fusiform gyrus, and insula   **MVPA:**  RSA:   - Bilateral IPS in both 2^nd^ and 5^th^ grades represented fraction magnitude to a similar degree - Bilateral IPS encoded absolute distance for nonsymbolic, symbolic, and mixed |
| Schmithorst & Brown, 2004 | fMRI, univariate analysis | - Independent component analysis with fraction addition and subtraction | **Independent component analysis:**   - Both fraction addition and subtraction associated with activity in the IPS, fusiform gyrus, anterior insula and middle frontal gyrus |
| Sprute, 2013 | fMRI, univariate analysis and MVPA | - Fractions vs. decimals vs. whole numbers | **Univariate analyses:**  Fractions vs. decimals :   - Fractions elicited greater response than decimals in the intraparietal sulcus (IPS), rostrolateral prefrontal cortex, inferior frontal cortex, cingulate gyrus, and bilateral cerebellum   Fractions vs. whole numbers:   - left inferior occipital and temporal gyri, and the right inferior and middle occipital gyri   Conjunction of fractions, decimals, and whole numbers:   - IPS, superior parietal lobule (SPL), right middle occipital gyrus, and left precentral gyrus in the ventral premotor area   **MVPA:**  Classification within the IPS:   - Accuracy above 63% if discriminating fractions, decimals, and whole numbers, with higher accuracy in the right IPS than the left. Accuracy reached 80% when discriminating fractions vs. whole numbers   Whole brain searchlight:   - Fractions vs. decimals vs. whole numbers: Bilateral IPS, bilateral lingual, inferior occipital, and middle occipital gyri, left fusiform, the left inferior and superior   temporal gyri, and the right middle temporal gyrus, bilateral precentral, inferior, middle, and superior frontal  gyri, cingulate gyrus, and cerebellum   - Decimals vs. whole numbers: right SPL, IPS, precuneus, lingual gyrus, inferior and middle occipital gyri, left inferior, middle, and superior temporal gyri and fusiform gyrus - Fractions vs. whole numbers: bilaterally in the IPS, SPL, inferior parietal lobule, precuneus, bilateral lingual, inferior occipital, and middle occipital gyri, right fusiform, left inferior, middle, and superior temporal gyri, bilateral precentral, medial, inferior, middle, and superior frontal gyri, and cingulate cortex |
| Starling-Alves, 2021 | fMRI, univariate analysis | - Near vs. far distances | **Univariate analyses:**  2^nd^ to 3^rd^ grade:   - Stronger intraparietal sulcus (IPS) activity for near than far distances for with nonsymbolic condition in 2^nd^ grade - No difference between near and far distances for fractions in 2^nd^ grade. - Stronger IPS activity for near than far distances in 3^rd^ grade, for both nonsymbolic ratios and fractions   5^th^ to 6^th^ grade:   - Stronger IPS and frontal regions activity for near than far distances for nonsymbolic ratios and fractions in 5^th^ and 6^th^ grades |
| Tzur & Depue, 2014 | fMRI, univariate analysis | - Fraction vs. whole number comparisons | **Univariate analyses:**  Fractions vs. whole-number:   - Bilateral IPS and angular gyrus, ventral visual processing stream, dorsal fronto-parietal control network, ventral-frontal working memory network & pulvinar, and the supplementary motor area |
| Wortha et al., 2020 | fMRI, univariate analysis | - Near vs. far distances - Before vs. after training | **Univariate analyses:**  Before training: near vs. far distance:   - Fraction vs. line ratio: intraparietal cortex (hIP3), superior parietal lobule (SPL), inferior frontal gyrus (IFG; Areas 44 and 45), bilateral inferior temporal gyri as well as bilateral insula - Line ratio vs. line ratio: right-hemispheric fronto-parietal network centered around the right intraparietal sulcus (hIP3) and IFG - Fraction vs. fraction: n.s.   Before vs. after training:   - Fraction vs. line ratio: n.s. - Line ratio vs. line ratio: n.s. - Fraction vs. fraction: bilateral fronto-parietal network centered around the intraparietal sulcus (hIP3), right SPL, left inferior parietal lobe, right fusiform gyrus, bilateral frontal cortex, and the left thalamus |

**Table S2. Most commonly associated processing-related terms for each coordinate from neurosynth.org for each meta-analytic cluster.**

| **Cluster** | **Hemisphere** | **Region**  **(Brodmann’s area)** | **Center MNI coordinate** | | | **Most commonly associated terms** |
| --- | --- | --- | --- | --- | --- | --- |
|  |  |  | **x** | **y** | **z** |  |
| 1 | Left | Inferior parietal lobule (BA 40) | -33 | -55 | 46 | working memory, word, memory, reading, calculation, arithmetic |
| 2 | Left | Precentral gyrus  (BA 9) | -45 | 12 | 31 | phonological, language, semantic, reading, word, verbal |
| 3 | Bilateral | Medial frontal gyrus (BA 6) | -2 | 22 | 46 | memory, words, working memory, phonological, orthographic, retrieval |
| 4 | Right | Precuneus  (BA 7) | 28 | -65 | 46 | calculation, arithmetic, visual, reading, shapes, attention |
| 5 | Right | Middle frontal gyrus (BA 9) | 46 | 33 | 23 | working memory, executive, memory, interference, task difficulty |
| 6 | Right | Inferior parietal lobule (BA 40) | 42 | -45 | 48 | working memory, memory load, visuospatial, calculation, attentional, memory |
| 7 | Right | Lingual gyrus (BA 17) | 15 | -92 | -3 | visual, working, reading, object, abstract |
| 8 | Right | Insula (BA 13) | 33 | 26 | 0 | working memory, orthographic, demands, task difficulty, memory |

*Note.* Excludes generic terms (e.g., "task"", ""processing", "related") and anatomical terms (e.g., "parietal", "ifg", "frontal")

**Table S3. Sensitivity analysis: ALE meta-analytic results for functional MRI studies investigating fraction near distance vs. fraction far distance**

| **Cluster** | **Hemisphere** | **Region**  **(Brodmann’s area)** | **Center MNI coordinate** | | | **Cluster size (mm^3^)** | **ALE value** | **Z** |
| --- | --- | --- | --- | --- | --- | --- | --- | --- |
|  |  |  | **x** | **y** | **z** |  |  |  |
| 1 | Left | Medial frontal gyrus | -4 | 24 | 4 | 1336 | 0.017 | 5.20 |
| 2 | Right | Precentral gyrus  (BA 9), middle frontal gyrus | 46 | 32 | 29 | 1040 | 0.013 | 4.33 |
| 3 | Right | Inferior parietal lobule | 40 | -48 | 46 | 584 | 0.018 | 5.36 |

*Note.* Cluster-level family-wise error (FWE) thresholded at p < .05, with a voxel-level threshold of p < .001 and 1,000 permutations. *Indicates in how many leave-one-study-out iterations (max = 7) the cluster was still identified in the ALE meta-analysis. A larger number indicates more robust findings.

**TableS4. Sensitivity analysis: ALE meta-analytic results for functional MRI studies investigating fraction vs. natural number contrasts**

| **Cluster** | **Hemisphere** | **Regions**  **(Brodmann’s area)** | **Center MNI coordinate** | | | **Cluster size (mm^3^)** | **ALE value** | **Z** |
| --- | --- | --- | --- | --- | --- | --- | --- | --- |
|  |  |  | **x** | **y** | **z** |  |  |  |
| 1 | Left | Superior parietal lobule, Precuneus, angular gyrus | -27 | -62 | 49 | 1986 | 0.020 | 5.32 |
| 2 | Left | Precentral gyrus  (BA 9), Inferior Frontal gyrus, middle frontal gyrus | -45 | 9 | 31 | 2096 | 0.016 | 4.56 |
| 3 | Right | Cerebellum | 14 | 78 | -21 | 736 | 0.019 | 5.08 |
| 4 | Right | Precuneus  (BA 7) | 28 | -68 | 46 | 1040 | 0.016 | 4.62 |

*Note.* Cluster-level family-wise error (FWE) thresholded at p < .05, with a voxel-level threshold of p < .001 and 1,000 permutations. *Indicates in how many leave-one-study-out iterations (max = 7) the cluster was still identified in the ALE meta-analysis. A larger number indicates more robust findings.
